# *AS3MT* Polymorphism: A Risk Factor for Epilepsy Susceptibility and Adverse Drug Reactions to Valproic Acid and Oxcarbazepine Treatment in Children From South China

**DOI:** 10.3389/fnins.2021.705297

**Published:** 2021-11-26

**Authors:** Xiaomei Fan, Yuna Chen, Jieluan Lu, Wenzhou Li, Xi Li, Huijuan Guo, Qing Chen, Yanxia Yang, Hanbing Xia

**Affiliations:** ^1^Shenzhen Baoan Women’s and Children’s Hospital, Jinan University, Shenzhen, China; ^2^College of Pharmacy, Jinan University, Guangzhou, China; ^3^Shenzhen Nanshan District Shekou People’s Hospital, Shenzhen, China

**Keywords:** pediatric epilepsy, AS3MT, gene polymorphism, susceptibility, adverse drug reaction, valproic acid, oxcarbazepine

## Abstract

Epilepsy is a common neurologic disorder characterized by intractable seizures, involving genetic factors. There is a need to develop reliable genetic markers to predict the risk of epilepsy and design effective therapies. Arsenite methyltransferase (AS3MT) catalyzes the biomethylation of arsenic and hence regulates arsenic metabolism. *AS3MT* variation has been linked to the progression of various diseases including schizophrenia and attention deficit or hyperactivity disorder. Whether genetic polymorphism of *AS3MT* contributes to epilepsy remains unclear. In this study, we investigated the association of *AS3MT* gene polymorphism with susceptibility to epilepsy in children from south China. We also explored the effect of *AS3MT* variation on the safety of antiepileptic drugs. Genotypic analysis for *AS3MT* rs7085104 was performed using samples from a Chinese cohort of 200 epileptic children and 244 healthy individuals. The results revealed a genetic association of *AS3MT* rs7085104 with susceptibility to pediatric epilepsy. Mutant homozygous GG genotype exhibited a lower susceptibility to childhood epilepsy than AA genotype. Carriers of *AS3MT* rs7085104 AA genotype exhibited a higher risk of digestive adverse drug reactions (dADRs) in children when treated with valproic acid (VPA) or oxcarbazepine (OXC). Additionally, bioinformatics analysis identified eight *AS3MT* target genes related to epilepsy and three *AS3MT*-associated genes in VPA-related dADRs. The effects of AS3MT on epilepsy might involve multiple targets including *CNNM2*, *CACNB2*, *TRIM26*, *MTHFR*, *GSTM1*, *CYP17A1*, *NT5C2*, and *YBX3*. This study reveals that *AS3MT* may be a new gene contributing to epileptogenesis. Hence, analysis of *AS3MT* polymorphisms will help to evaluate susceptibility to pediatric epilepsy and drug safety.

## Introduction

Epilepsy is a common chronic neurological disease among children. It is characterized by unpredictable lasting epileptic seizures and numerous serious neurobiological, cognitive, and psychosocial consequences and a high financial burden, therefore it is a major health concern worldwide (Beghi et al., 2005; Fisher et al., 2014). The overall risk of death in epileptic children is approximately ten times higher compared with the general population (Donner et al., 2017). Approximately 35 in every 100,000 Chinese children suffer epilepsy annually (Hu et al., 2015). Advances in neonatal and critical disease care during the last several decades have reduced premature mortality, thus resulting in increasing cases of pediatric epilepsy. Epilepsy etiology involves genetics, metabolism, infection, immunity, structure and unknown factors (Abramovici and Bagić, 2016; Scheffer et al., 2017). Antiepileptic medications are the main treatment strategies for epilepsy, and act by suppressing the seizures rather than modifying the disease process. Non-pharmacological approaches include curative surgery, palliative surgical procedures, and ketogenic diet. Epilepsy is regarded as a symptom condition with multiple risk factors and high genetic predisposition (Thijs et al., 2019). Since identification of CHRNA4 gene, the first epilepsy gene in 1995 (Steinlein et al., 1995), several studies have focused on developing reliable and potential markers of genetic polymorphism related to epilepsy. Currently, more than 500 epilepsy-associated genes have been identified (Wang et al., 2017). However, genetic basis of epilepsy has not been fully explored, therefore, further studies on epilepsy genomics are needed.

Arsenite methyltransferase (AS3MT) catalyzes transfer of a methyl group from *S*-adenosyl-L-methionine (SAMe) to trivalent arsenic resulting in biomethylation of arsenic *in vivo* and *in vitro*, thus it plays a critical role in arsenic metabolism (Lin et al., 2002; Liu et al., 2020). *AS3MT* (previously called *CYT19*) gene variation affects arsenic methylation activity in children and adults (Antonelli et al., 2014; De Loma et al., 2018). *AS3MT* gene is associated with various diseases including Borst-Jadassohn intraepidermal carcinoma of dermal system, liver injury, schizophrenia, and attention deficit or hyperactivity disorder (Li et al., 2016; Zhao et al., 2020). Previous studies indicated that the *AS3MT* gene was ubiquitously expressed in the mouse brain (cerebral cortex, hippocampus, and cerebellum) and adult human neurons and astrocytes, and upregulated during human stem cell differentiation toward neuronal fates (Sánchez-Peña et al., 2010; Li et al., 2016). A two-stage GWAS of schizophrenia with large sample sizes in the Han Chinese population showed a significant association for AS3MT 10q24.32 loci (Yu et al., 2017). *AS3MT* rs7085104 was a schizophrenia-associated risk implicated in alteration of striatal dopamine synthesis capacity (Duarte et al., 2016; D’Ambrosio et al., 2019). Schizophrenia is associated with epilepsy based on neuropathological, clinical, and epidemiological data and at genetic level (Vonberg and Bigdeli, 2016). However, studies have not explored the role of the gene mutation of *AS3MT* in epilepsy.

The aim of this study was to explore the role of *AS3MT* rs7085104 genetic polymorphism on the risk of pediatric epilepsy in southern Chinese children. Target pathway/function network of *AS3MT* in epilepsy was explored through bioinformatic analysis to predict underlying mechanisms. Furthermore, the role of *AS3MT* rs7085104 variation on adverse drug reactions (ADRs) associated with antiepileptic drugs (AEDs) was evaluated.

## Materials and Methods

### Subjects

Pediatric patients with epilepsy (*n* = 200) and healthy individuals (*n* = 244) of Han Chinese descent from Shenzhen Baoan Women’s and Children’s Health Hospital were enrolled into this case-control study as described previously (Fan et al., 2020). All participants or their parents signed an informed consent approved by Baoan Women’s and Children’s Health Hospital ethics committee. Patients aged <16 years and >1 month were diagnosed with definitely characterized epilepsy or epileptic syndrome by two independent experienced clinicians following the latest updated version of International League Against Epilepsy (ILAE) commission’s classification (Scheffer et al., 2017). Detailed clinical data of all patients including basic demographic characteristics, epilepsy classification, age of seizure onset, family history, history of status epilepticus, abnormal delivery or delayed discharge from NICU, and therapeutic drugs were retrieved from the hospital information system (HIS) and through telephone follow-up and were analyzed retrospectively. Patients without a clear diagnosis of epilepsy and those with non-reliable seizure frequency, a history of pseudoseizures, or presence of any diseases that may aggravate epilepsy (*n* = 39) were excluded from this study. Participants in the control group were individuals with similar age with patients in the case group and were clinically free of epilepsy or had no family history of central nervous system disorders. Children missing the above-mentioned clinical data were excluded from this study.

Patients with pediatric epilepsy who had received one or more AEDs for at least 6 months were used for pharmacogenetic studies to explore the correlation between AS3MT and ADRs. Patients with incomplete medication records were excluded. Patients who developed AEDs-associated ADRs whose causative drugs could not be established were also excluded. Each ADR report was analyzed by two authors using the WHO-UMC causality assessment scale.

### SNP Selection and Genotyping

Peripheral venous blood samples were collected from 444 children during their regular outpatient visits to Baoan Women’s and Children’s Health Hospital. Genomic DNA was extracted from whole blood following the protocol described previously (Loparev et al., 1991). DNA samples were amplified using polymerase chain reaction (PCR). Genotyping primers of *AS3MT* rs7085104 used in this study were as follows: forward primer sequence: 5′-ACGTTGGATGTGGTCTCCGTTTTGGTGATG-3′; reverse primer sequence: 5′-ACGTTGGATGCACATTC CGTGAAATCCATC-3′ and extended primer sequence: 5′-TGTGCAGTTCTGTGAACTC-3′. The PCR reaction system comprised 10 ng template DNA and 0.5 μM of primers. PCR reaction was performed under the following conditions: initial denaturation set at 95°C for 2 min followed by 45 cycles of denaturation at 95°C for 30 s, annealing at 56°C for 30 s and extension at 72°C for 1 min followed by a final extension of 72°C for 5 min. PCR products were purified by shrimp alkaline phosphatase (SAP) treatment. The cycle sequencing reaction was then performed using Complete iPLEX^®^ Gold Genotyping Reagents (Agena Bioscience™, San Diego, CA, United States). Genotyping of polymorphism was performed with Sequenom MassArray platform (Agena Bioscience, San Diego, CA, United States) and iPLEX Gold Assay. MassArray system comprises MassArray Analyzer mass spectrometer and integrated data analysis software.

### Analysis of Protein–Protein Interaction Network and Functional Enrichment Analysis

Protein function of AS3MT was explored through protein–protein interaction (PPI), which elucidates the role of regulation among proteins (Vella et al., 2017). STRING database v11.0^1^, a public database containing interactions between known and predicted proteins (Szklarczyk et al., 2018), was used to construct PPI network of AS3MT. The minimum required interaction score was set as medium confidence (0.400) and the maximum number of interactors was less than 50 (1st shell not more than 50). The PPI network was further visualized using Cytoscape v3.6.0 to present AS3MT-related genes. Metascape was used for GO analysis and Reactome pathway analysis of potential targets of AS3MT in the present study. *P*-value < 0.01, minimum count of 3, and enrichment factor >1.5 were used for filtering terms. Membership similarity (Kappa score) > 0.3 was applied as the threshold to group which enriched terms into clusters. A subset of representative terms was then selected from the cluster and used to construct a network. Each term was presented as a circle node, and terms with a similarity score > 0.3 were linked edges. The network was visualized using Cytoscape (v3.6.0) software.

### Potential Targets of *AS3MT* in Epilepsy or Digestive Adverse Drug Reactions

To further explore the identified disease-related genes, GeneCards^2^ and DisGeNET^3^ databases were used to retrieve epilepsy-associated genes and digestive diseases-associated genes. Genes obtained were further filtered based on the number of pubmed IDs (PMIDs) > 0 for DisGeNET and the relevance score ≥ 1 for GeneCards. To obtain potential targets of *AS3MT* in epilepsy or dADRs, genes related to AS3MT identified in PPI analysis were intersected with epilepsy-associated genes and digestive diseases-associated genes from DisGeNET and GeneCards databases.

### Statistical Analysis

All statistical analyses were performed using the Statistical Package for Social Sciences (SPSS) software (V25.0). Differences in age, gender, and AEDs therapy distribution between cases and controls were analyzed using Mann--Whitney *U* test and Chi-square test by preliminary cross-tabulations. SNPStats is a web-based tool designed for analyzing association studies. SNPStats Web Tool^4^ was used to calculate genotype distribution and minor allelic frequency using Exact test Hardy–Weinberg equilibrium calculations for *AS3MT* rs7085104 SNP in case and control groups. Data on SNPs’ allele contrast and five genetic inheritance models (codominant, dominant, recessive, overdominance, and additive genetic correlation) between the two groups were also analyzed. Odds ratio (OR) values and relative risk (95% CI) were calculated for multiple testing. Values with *p* < 0.05 were considered statistically significant. The power of this study for the minimum sample size calculation was computed for verification as described in our previous study (Fan et al., 2020). Power > 0.9 was obtained based on the frequency distribution of three *AS3MT* rs7085104 genotypes from participants in the control and case groups.

## Results

### Clinical Characteristics of Epileptic Children

General clinical characteristics of all the participants including 200 cases and 244 controls in this study were analyzed. The case group comprised 85 (42.5%) females and 115 (57.5%) males with an average age of 2.99 ± 3.09 years at the first seizure onset and 3.4 ± 3.1 years at the time diagnosis was confirmed. The control group consisted of 112 (45.9%) females and 132 (54.1%) males with a mean age of 4.6 ± 2.7 years. Analysis showed that there were no significant differences in gender between the two groups. Twenty-three epileptic children at their perinatal stage experienced diseases like hypertensive disorders, preeclampsia and eclampsia, gestational diabetes and threatened abortion. Twenty-three children experienced abnormal delivery or delayed discharge from NICU with invasive mechanical ventilation owing to severe diseases. Forty-four children with epilepsy suffered febrile seizure and twenty patients had a history of status epilepticus. The family members of 31 children suffered from febrile seizures, epilepsy, inherited metabolic diseases, and chromosome abnormality, among whom ten children experienced febrile seizure.

**FIGURE 1:**
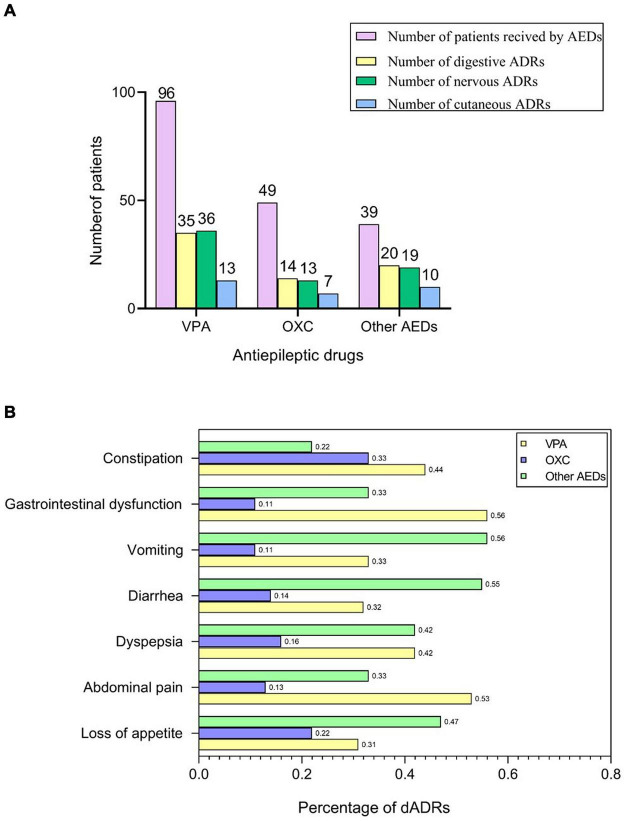
AEDs related-ADRs observed in our study. **(A)** Number of patients who received AEDs and presented with ADRs. **(B)** Frequency of dADRs in epileptic children.

AEDs used in our cases were VPA (*n* = 96, 52.2%), OXC (*n* = 49, 26.6%), and other AEDs (*n* = 39, 21.2%), which was consistent with ILAE guidelines (Glauser et al., 2013; Wilmshurst et al., 2015). Among 174 pediatric patients with epilepsy receiving AEDs, 123 children presented with ADRs including digestive adverse drug reactions (dADRs), skin reactions and neurological adverse reactions. Causality assessment using WHO UMC scale showed 3.7% certain, 85.9% probable, and 10.4% possible association of ADRs with AEDs. The percentage of patients presenting with ADRs was significantly higher compared with those without ADRs (64.55% vs. 35.45% for monotherapy and 81.25% vs. 18.75% for polytherapy). Polytherapy of AEDs resulted in more nADRs and cADRs compared with monotherapy (Supplementary Table S1). This finding implied that polytherapy highly predisposed children with epilepsy to ADRs compared with monotherapy (Table 1). The number of patients who presented with dADRs, nervous ADRs (nADRs), and cutaneous ADRs (cADRs) was 69 (39.7%), 58 (33.3%), and 30 (17.2%), respectively, mainly triggered by VPA and OXC (Figure 1A). Loss of appetite, dyspepsia, abdominal pain, diarrhea, and vomiting were the main manifestations of AEDs related-dADRs (Figure 1B). Number of pediatric patients with and without dADRs was significantly different in different groups based on age of diagnosis (OR = 2.11, 95%CI = 1.12–3.90, *p* = 0.0169) (Table 1). Age of diagnosis in patients with dADRs was 2.71 ± 2.55 years, which was significantly less than that of patients without dADRs (4.12 ± 3.21 years). The incidence rate of dADRs in patients with childhood epilepsy receiving VPA alone was higher than that of patients treated using VPA in combination with other AEDs.

**TABLE 1:** Clinical and demographic characteristics of patients with ADRs.

Characteristics	Cases (n, %)				Controls (n, %)				*P*-value			
	All ADRs	dADRs	VPA-dADRs	OXC-dADRs	ADRs-No	dADRs-No	VPA-dADRs-No	OXC-dADRs-No	^a^ *P*	^b^ *P*	^c^ *P*	^d^ *P*
**Age of diagnosis (years)**												
1 month–2	58 (47.2%)	39 (56.5%)	19 (54.3%)	5 (35.7%)	21 (41.2%)	40 (38.1%)	29 (47.5%)	13 (37.1%)	0.471	0.0169*	0.5247	0.9253
2–16	65 (52.8%)	30 (43.5%)	16 (45.7%)	9 (64.3%)	30 (58.8%)	65 (61.9%)	32 (52.5%)	22 (62.9%)				
Mean ± SD	3.53 ± 3.05	2.71 ± 2.55	3.01 ± 2.89	3.37 ± 2.38	3.61 ± 2.99	4.12 ± 3.21	3.58 ± 3.23	4.15 ± 3.55	0.8364	0.0026*	0.9476	0.8457
**Gender**												
Male	69 (56.1%)	37 (53.6%)	17 (48.6%)	5 (35.7%)	29 (56.9%)	61 (58.1%)	38 (62.3%)	16 (45.7%)	0.9262	0.5607	0.1907	0.5228
Female	54 (43.9%)	32 (46.4%)	18 (51.4%)	9 (64.3%)	22 (43.1%)	44 (41.9%)	23 (37.7%)	19 (54.3%)				
**AEDs therapy**												
Monotherapy	71 (57.7%)	50 (72.5%)	22 (62.9%)	8 (57.1%)	39 (76.5%)	70 (66.7%)	25 (41.0%)	24 (68.6%)	0.0196*	0.2677	0.0391*	0.4477
Polytherapy	52 (42.3%)	19 (27.5%)	13 (37.1%)	6 (42.9%)	12 (23.5%)	35 (33.3%)	36 (59.0%)	11 (31.4%)				
**Causality assessment** ^e^												
Certain	10 (3.7%)	2 (1.6%)	2 (4.0%)	0		8 (5.6%)						
Probable	232 (85.9%)	112 (88.9%)	44 (88.0%)	17 (80.9%)		120 (83.3%)						
Possible	28 (10.4%)	12 (9.5%)	4 (8.0%)	4 (19.1%)		16 (11.1%)						

*^a^P-value obtained from patients with ADRs group (n = 123) versus patients without ADRs group (n = 51).*

*^b^P-value obtained from patients with dADRs group (n = 69) versus patients without dADRs group (n = 105).*

*^c^P-value obtained from patients with dADRs induced by VPA group (n = 35) versus patients without dADRs induced by VPA group (n = 61).*

*^d^P-value obtained from patients with dADRs induced by OXC group (n = 14) versus patients without dADRs induced by OXC group (n = 35).*

*^e^The number of ADRs observed in epileptic children receiving AEDs.*

*P < 0.05.

### The Polymorphism of *AS3MT* Gene Associated With Potential Risks of Pediatric Epilepsy

Frequency distribution of *AS3MT* rs7085104 genotype in cases and controls was shown in Table 2. Significant differences of frequency distribution between the two groups were observed. Allele analysis showed that allele A had a higher frequency among epileptic patients compared with that in healthy children (51.8% and 41.2%, respectively). Allele frequency of *AS3MT* rs7085104 in our study was consistent with that from the HapMap study. Distribution of AA, GA, and GG genotypes in the case group was 28.0%, 47.5%, and 24.5%, respectively, whereas the distribution of these genotypes in the control group was 16.8%, 48.8%, and 34.4% (*p* = 0.0068). Among three *AS3MT* rs7085104 genotypes, heterozygous GA genotype exhibited the highest frequency of distribution in both case group and control group. Carriers of two polymorphic *AS3MT* rs7085104 A alleles (mutant homozygous AA genotype) showed a higher epilepsy risk of childhood epilepsy (OR = 1.93, 95% CI = 1.22–3.04, *p* = 0.0046). However, mutant homozygous GG genotype exhibited lower susceptibility to childhood epilepsy compared with AA genotype (AA *vs.* GG: OR = 1.52, 95% CI = 1.16–1.98, *p* = 0.0019). These results indicated that children carrying AA diplotype of *AS3MT* rs7085104 had higher risk of epilepsy.

**TABLE 2:** Comparison of *AS3MT* rs7085104 diplotype distribution between epileptic children and healthy children.

Genetic model	Diplotype	Cases (*n* = 200)	Controls (*n* = 244)	OR (95% CI)	*p*-value
Allele contrast	A *vs.* G	207 (51.8%)/ 193 (48.2%)	201 (41.2%)/ 287 (58.8%)	1.00 1.53 (1.17–2.00)	0.0017**
Codominant	AA *vs.* GA *vs.* GG	56 (28.0%)/ 95 (47.5%)/ 49 (24.5%)	41 (16.8%)/ 119 (48.8%)/ 84 (34.4%)	1.00 1.71 (1.05–2.78) 2.34 (1.37–4.00)	0.0068**
Dominant	AA *vs.* GA + GG	56 (28.0%)/ 144 (72.0%)	41 (16.8%)/ 203 (83.2%)	1.00 1.93 (1.22–3.04)	0.0046**
Recessive	AA + GA *vs.* GG	151 (75.5%)/ 49 (24.5%)	160 (65.6%)/ 84 (34.4%)	1.00 1.62 (1.07–2.45)	0.022*
Overdominant	AA + GG *vs.* GA	105 (52.5%)/ 95 (47.5%)	125 (51.2%)/ 119 (48.8%)	1.00 1.05 (0.72–1.53)	0.79
Log-additive	AA *vs.* GG	56 (28.0%)/ 49 (24.5%)	41 (16.8%)/ 84 (34.4%)	1.00 1.52 (1.16–1.98)	0.0019**

* p < 0.05,

** p < 0.01 versus control group.

Stratification analysis of *AS3MT* rs7085104 was further conducted using selected variables from these patients. Frequency distribution of *AS3MT* rs7085104 genotypes showed significant difference between cases and controls in the subgroup of female patients (*p* = 0.0021, Table 3). Epileptic patients with focal onset or generalized onset showed significant differences in genotype distribution of *AS3MT* rs7085104 compared with healthy individuals (*p* = 0.0005 and *p* = 0.0123, respectively). The proportions of AA genotype in patients with focal onset seizures and generalized onset seizures were significantly higher than those of the control group.

**TABLE 3:** Stratification analysis of *AS3MT* rs7085104 genotypes using selected variables in epileptic children and healthy children.

Variables	Cases			Controls			*p*-value
	AA	GA	GG	AA	GA	GG	
**Confirmed age (years)**							
1 month -2	28 (29.5%)	47 (49.5%)	20 (21.0%)	5 (14.7%)	20 (58.8%)	9 (26.5%)	0.24
2–16	29 (28.4%)	45 (44.1%)	28 (27.5%)	36 (17.1%)	99 (47.2%)	75 (35.7%)	0.056
**Gender**							
Male	29 (25.2%)	58 (50.4%)	28 (24.4%)	22 (19.6%)	54 (48.2%)	36 (32.2%)	0.36
Female	29 (34.1%)	36 (42.4%)	20 (23.5%)	19 (14.4%)	65 (49.2%)	48 (36.4%)	0.0021**
**Seizure classification**							
Focal onset	19 (40.4%)	12 (25.5%)	16 (34.1%)	41 (16.8%)	119 (48.8%)	84 (34.4%)	0.0005***
Generalized onset	36 (25.9%)	73 (52.5%)	30 (21.6%)				0.0123*
Unknown onset	3 (21.4%)	9 (64.3%)	2 (14.3%)				—

* p < 0.05,

** p < 0.01,

*** p < 0.001 versus control group.

### Polymorphism of *AS3MT* Gene Associated With Predisposition to Digestive Adverse Drug Reactions Induced by Valproic Acid and Oxcarbazepine

Frequency distribution of *AS3MT* rs7085104 genotypes in patients with and without ADRs in this study was further analyzed. Allele analysis showed that allele A was more frequent in epileptic children who suffered dADRs when receiving OXC than allele G (71.4% and 47.1%, respectively), and the frequency in patients without dADRs was on the contrary (Table 4). Carriers of *AS3MT* rs7085104 AA genotype showed a higher risk of dADRs after treatment with VPA or OXC (OR = 2.77, 95% CI = 1.12–6.86, *p* = 0.027 for VPA and OR = 4.50, 95% CI = 1.20–16.85, *p* = 0.023 for OXC) compared to those with GA and or GG genotypes. However, mutant homozygous GG genotype exhibited a lower predisposition to dADRs after administration with VPA or OXC drugs compared with AA genotype. These findings indicate that genetic polymorphism of *AS3MT* rs7085104 is correlated with the potential risk of dADRs caused by VPA and OXC. Furthermore, association of *AS3MT* rs7085104 polymorphism with other ADRs in central nervous system, skin and subcutaneous tissues was explored but no significant difference was observed (Supplementary Tables S2, S3).

**TABLE 4:** Comparison of *AS3MT* rs7085104 diplotype distribution for epileptic children with or without dADRs after receiving AEDs.

Drug	Genetic model	Diplotype	Patients with dADRs (*n* = 35)	Patients without dADRs (*n* = 61)	OR (95% CI)	*p*-value
VPA	Allele contrast	A *vs.* G	44 (62.9%)/ 26 (37.1%)	58 (47.5%)/ 64 (52.5%)	1.00 1.86 (1.03–3.35)	0.0508
	Codominant	AA *vs.* GA *vs.* GG	15 (42.9%)/ 14 (40.0%)/ 6 (17.1%)	13 (21.3%)/ 32 (52.5%)/ 16 (26.2%)	1.00 2.64 (1.00–6.98) 3.08 (0.93–10.18)	0.084
	Dominant	AA *vs.* GA + GG	15 (42.9%)/ 20 (57.1%)	13 (21.3%)/ 48 (78.7%)	1.00 2.77 (1.12–6.86)	0.027*
	Recessive	AA + GA *vs.* GG	29 (82.9%)/ 6 (17.1%)	45 (73.8%)/ 16 (26.2%)	1.00 1.72 (0.60–4.90)	0.3
	Overdominant	AA + GG *vs.* GA	21 (60.0%)/ 14 (40.0%)	29 (47.5%)/ 32 (52.5%)	1.00 1.66 (0.71–3.84)	0.24
	Log-additive	AA *vs.* GG	15 (42.9%)/ 6 (17.1%)	13 (21.3%)/ 16 (26.2%)	1.00 1.85 (1.01–3.40)	0.042*

**Drug**	**Genetic model**	**Diplotype**	**Patients with dADRs (*n* = 14)**	**Patients without dADRs (*n* = 35)**	**OR (95% CI)**	***p*-value**

OXC	Allele contrast	A *vs.* G	20 (71.4%)/ 8 (28.6%)	33 (47.1%)/ 37 (52.9%)	1.00 2.80 (1.08–7.19)	0.043*
	Codominant	AA *vs.* GA *vs.* GG	8 (57.1%)/ 4 (28.6%)/ 2 (14.3%)	8 (22.9%)/ 17 (48.6%)/ 10 (28.6%)	1.00 4.25 (0.98–18.40) 5.00 (0.82–30.46)	0.075
	Dominant	AA *vs.* GA + GG	8 (57.1%)/ 6 (42.9%)	8 (22.9%)/ 27 (77.1%)	1.00 4.50 (1.20–16.85)	0.023*
	Recessive	AA + GA *vs.* GG	12 (85.7%)/ 2 (14.3%)	25 (71.4%)/ 10 (28.6%)	1.00 2.40 (0.45–12.71)	0.28
	Overdominant	AA + GG *vs.* GA	10 (71.4%)/ 4 (28.6%)	18 (51.4%)/ 17 (48.6%)	1.00 2.36 (0.62–8.98)	0.19
	Log-additive	AA *vs.* GG	8 (57.1%)/ 2 (14.3%)	8 (22.9%)/ 10 (28.6%)	1.00 2.57 (1.01–6.57)	0.036*

* p < 0.05 versus epileptic patients without dADRs.

### Target Network of *AS3MT* in Epilepsy and Digestive Adverse Drug Reactions

**FIGURE 2:**
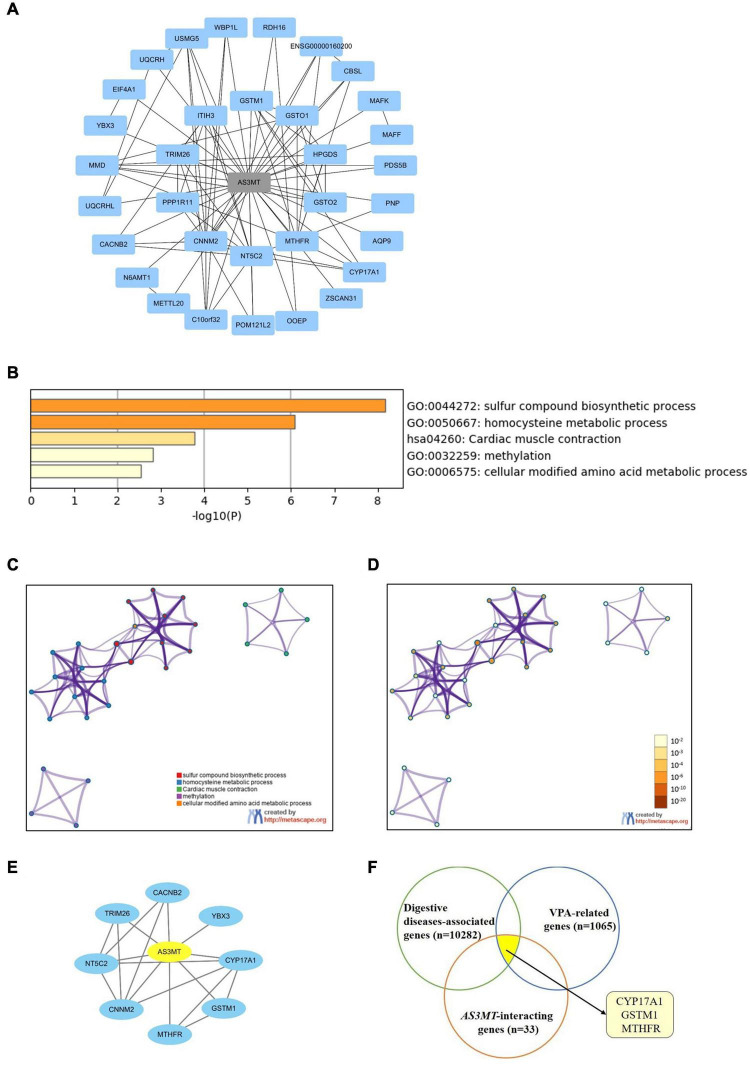
Network of AS3MT related genes implicated in epilepsy and VPA-related dADRs using bioinformatic analysis. **(A)** PPI network of AS3MT comprising 34 nodes and 86 edges. Each target protein was represented by a node. **(B)** Functional enrichment analysis of 33 target genes related with AS3MT. The top non-redundant enrichment clusters identified using Metascape and statistical significance of one per cluster represented by a discrete color scale. **(C,D)** Metascape visualization of the interactome network. Nodes are colored based on their identities **(C)** and *p*-values **(D)**. Each circle node represents a term, where its size is proportional to the number of input genes clustering into that term. The color represents cluster identity of the node. A darker color represents high statistical significance of the node. **(E)** PPI network between AS3MT and 8 common genes associated with epilepsy comprising 9 nodes and 18 edges. **(F)** Three common genes identified for AS3MT in VPA-related dADRs.

STRING database was used to explore the functional relationships and interactions of *AS3MT*. Thirty-three genes were found to interact with *AS3MT*. A PPI network comprising 34 nodes and 86 edges was constructed (Figure 2A). These genes were implicated in a total of 154 pathways and functions and were mainly involved in “sulfur compound biosynthetic process,” “homocysteine metabolic process,” “cardiac muscle contraction,” “methylation,” and “cellular modified amino acid metabolic process”, which were the top five clusters based on *p*-values (Figures 2B–D). Furthermore, 1176 genes from DisGeNET and 5064 genes from GeneCards database were shown to be associated with epilepsy. After removing the duplicates from two databases, a total of 5166 epilepsy-associated genes were obtained. These genes were intersected with 33 genes related to *AS3MT* from PPI analysis. Eight common genes including *MTHFR*, *GSTM1*, *CYP17A1*, *NT5C2*, *YBX3*, *CNNM2*, *CACNB2*, and *TRIM26* were identified as potential targets of *AS3MT* in epilepsy. *CNNM2*, *NT5C2*, and *TRIM26* were the top three key genes related to *AS3MT* according to high-ranking interaction score. The PPI network between eight targets and *AS3MT* is shown in Figure 2E. In addition, 10282 digestive diseases-associated genes from GeneCards and DisGeNET, 1065 VPA-related genes, and 116 OXC-related genes from DrugBank and SwissTargetPrediction were obtained. Thirty-three interacting genes of *AS3MT* were further intersected with digestive diseases-associated genes and VPA or OXC-related genes. Three common genes including CYP17A1, GSTM1, and MTHFR were identified for *AS3MT* in VPA-related dADRs (Figure 2F), however, no common gene was identified in OXC-related dADRs. These results indicate that AS3MT plays a role in epilepsy and AEDs-related ADRs likely through multiple targets.

## Discussion

Epilepsy, a common neurologic disease, has a genetic component contributed by multiple genes (Wang et al., 2017; Thijs et al., 2019). More than half of all epilepsy cases have a genetic basis. A major challenge on elimination of epilepsy is the inherent complexity and heterogeneity of known epileptic syndromes and differential genetic susceptibilities exhibited by patients at risk. Genetic testing as a routine diagnostic strategy of epilepsy can be effective for diagnosis and for guiding treatment (Pal et al., 2010). Mutations of several genes have been reported to exert a significant effect on susceptibility to idiopathic epilepsies and associated comorbidities (Canavati et al., 2019; Fan et al., 2020; Song et al., 2020). The largest genome-wide association study (GWAS) in epilepsy published by ILAE Consortium revealed sixteen genome-wide significant loci with eleven novel loci (Abou-Khalil et al., 2018; Perucca et al., 2020).

*AS3MT* (previously called *CYT19*) gene located in 10q24.32, encodes a 43 kDa cytosolic protein which is a cysteine rich enzyme that transfers a methyl group from *S*-adenosyl-L-methionine to trivalent arsenical (Lin et al., 2002; Liu et al., 2020). AS3MT plays an important role in catalysis of biomethylation of arsenic *in vivo* and *in vitro*. Previous studies reported that arsenic exposure had a toxic effect on the nervous system, causing nervous system dysfunction and psychosis such as polyneuropathy, EEG abnormalities and, in extreme cases, hallucinations, disorientation and agitation (Rodríguez et al., 2003). Genetic factors such as gene polymorphisms related to arsenic metabolism are used to determine arsenic concentrations in the human body (Chernoff et al., 2020). Arsenic methylation efficiency in children and adults is affected by *AS3MT* variation (Antonelli et al., 2014; De Loma et al., 2018). *AS3MT* is mainly expressed in human adrenal glands, liver, heart, kidney, and brain (Su et al., 2002). Moreover, *AS3MT* expression is highly expressed in adult human neurons and astrocytes during human stem cell differentiation toward neuronal fates and in brains of patients with schizophrenia compared with controls (Li et al., 2016). *AS3MT* gene was associated with schizophrenia and attention deficit or hyperactivity disorder (Li et al., 2016; Zhao et al., 2020). Notably, *AS3MT* rs7085104 as a schizophrenia-associated risk SNP altered striatal dopamine synthesis capacity (Duarte et al., 2016; D’Ambrosio et al., 2019; Cai et al., 2021). Dopamine pathway was regarded as a new regulating mechanism for epilepsy. There was a positive genetic correlation between schizophrenia and epilepsy (Vonberg and Bigdeli, 2016). Whether the SNP of *AS3MT* rs7085104 played a role in pediatric epilepsy remained unknown and thus we aimed to explore it. The findings of our study showed that frequency distribution of *AS3MT* rs7085104 genotypes was associated with pediatric epilepsy in a Han Chinese population from Southern China. Allele A showed higher frequency in children with epilepsy compared with healthy controls. Carries of *AS3MT* rs7085104 AA genotype showed a higher epilepsy risk, whereas children with GG genotype were less predisposed to epilepsy. The human-specific variable number of tandem repeat (VNTR) and *AS3MT* rs7085104 were significantly associated with *AS3MT*^*d*2*d*3^ mRNA expression in brains of Han Chinese donors with the A allele at rs7085104 predicting higher mRNA levels of this isoform (Cai et al., 2021). Additionally, another SNP rs10883795 was found to be associated with AS3MT expression levels and the mRNA levels of nearby genes, including CNNM2 and NT5C2 (Yu et al., 2017).

Use of antiepileptic drugs is the main treatment strategy for pediatric epilepsy. VPA, OXC, levetiracetam (LEV), and lamotrigine (LTG) are the main AEDs used for treatment of epilepsy in children. Children are generally at higher risk of toxic effects compared with adults. In our study, high incidences of ADRs associated with AEDs including dADRs, cADRs, and nADRs were observed. Notably, VPA was associated with most ADRs observed in this study, which was consistent with previous reports (Buchanan, 1992; Kaushik et al., 2019). Polytherapy significantly increased risk of ADRs in children. AEDs therapy in pediatric patients requires attention on safety and tolerability from clinicians. Knowledge of different mechanisms of AEDs-related adverse effects is essential to ensure safe treatment of pediatric epilepsy. Pharmacogenomics is a valuable tool for exploring association of inter-individual genetic variations with treatment response in children. *AS3MT* gene polymorphism was implicated in pediatric epilepsy, therefore, the role of gene mutation in responsiveness and safety of AEDs was further explored in this study. *AS3MT* rs7085104 polymorphism was correlated with potential risk of AEDs-induced dADRs. In addition, AA genotype predisposed pediatric children to dADRs after treatment with VPA or OXC.

Potential mechanisms of AS3MT in epilepsy were further explored through bioinformatics analysis. Eight common genes between *AS3MT* and epilepsy were identified. Out of the eight genes, *CNNM2*, *NT5C2*, and *TRIM26* were the top three key genes associated with *AS3MT*. *NT5C2* and *CNNM2* are involved in etiology and pathogenesis of schizophrenia and have been confirmed as schizophrenia susceptibility genes (Duarte et al., 2016; Guan et al., 2016). *NT5C2* encodes a phosphatase involved in cellular purine metabolism, which is associated with disorders characterized by psychiatric and psychomotor disturbances (Duarte et al., 2019). NT5C2 has a significantly high affinity for adenosine monophosphate and is involved in the extensive transcriptional programming which regulates cell maintenance, proliferation, migration, and differentiation during neurodevelopment (Itoh, 2013). Cyclin M2 (CNNM2) encodes a member of the protein family containing a cyclin box motif. It plays a role in brain development, neurological functioning and Mg^2+^ homeostasis. Mutations in *CNNM2* gene are implicated in mental retardation and seizures in patients with hypomagnesemia (Arjona et al., 2014; Accogli et al., 2019). The tripartite motif-containing 26 (TRIM26), localized in cytoplasmic bodies, comprises three zinc-binding domains, including RING, B-box type 1 and B-box type 2 domains, and a coiled-coil region, which have DNA-binding activity. TRIM26-associated diseases include neural tube defects and its related pathways are implicated in interferon gamma signaling and innate immune system. TRIM26 is associated with pathogenesis of schizophrenia (de Jong et al., 2012). Other common genes including *MTHFR*, *GSTM1*, *CYP17A1*, and *CACNB2* are implicated in neurological disorders such as epilepsy (Scher et al., 2011; Glister et al., 2012; Chbili et al., 2014; Kim and State, 2014). Furthermore, due to lack of direct evidence for AS3MT on dADRs, bioinformatics analysis was performed to explore the relationship between AS3MT and dADRs induced by VPA or OXC drugs. CYP17A1, GSTM1, and MTHFR were identified as the common genes of *AS3MT* in VPA-related dADRs whereas no common genes were identified in OXC-related dADRs. It was found that the *CYP17A1* rs743572 was in strong linkage disequilibrium (LD) with the *AS3MT* SNPs and had similar effects on the metabolite profiles (Schläwicke Engström et al., 2009; Gomez-Rubio et al., 2010). Previous studies reported that GSTM1 and MTHFR were associated with adverse reactions of VPA (Roy et al., 2008; Fricke-Galindo et al., 2018). Therefore, we deduce that AS3MT may exert its effect on epilepsy and VPA-induced dADRs through these genes.

Our study population included epileptic children with different types of and etiologies. Due to the relatively small sample size, the association between AS3MT and the specific epilepsy subtypes was not explored and no significant difference was observed for ADRs from other systems in our study. In further study, we will increase the sample size and further explore the role of *AS3MT* in different epilepsy subtypes and other AEDs related ADRs. Our study only investigated single SNP, which underestimate moderate and large effect sizes for epilepsy with numerous risk SNPs due to non-collapsibility of the odds ratio. The polygenic model of epilepsy and the LD between AS3MT with other nearby genes will be further investigated in genomic association studies. Furthermore, findings from bioinformatics analysis lack experimental validation. Therefore, *in vitro* and *in vivo* studies should be performed to elucidate the underlying mechanisms of *AS3MT* in epilepsy and dADRs. In addition, gene polymorphism may possibly alter gene transcription, therefore, *AS3MT* mRNA and protein expression in different genotypes of *AS3MT* rs7085104 should be determined to provide insights into the molecular mechanisms of AS3MT in pediatric epilepsy and drug safety.

## Conclusion

Epilepsy, one of the most common serious brain conditions, is a disease with complex symptoms, multiple risk factors, and a strong genetic component. Our study revealed that genetic polymorphism of *AS3MT* rs7085104 was associated with the susceptibility to pediatric epilepsy and risk of AEDs-related dADRs. Mutant homozygous GG genotype exhibited a lower susceptibility to childhood epilepsy than AA genotype. Carriers of *AS3MT* rs7085104 AA genotype tended to have a higher risk of dADRs for children after treatment with VPA or OXC. Bioinformatics analysis revealed that the effects of AS3MT on epilepsy likely involved multiple targets including *MTHFR*, *GSTM1*, *CYP17A1*, *NT5C2*, *YBX3*, *CNNM2*, *CACNB2*, and *TRIM26*. *AS3MT* may be a novel potential gene implicated in pathogenesis of epilepsies. Therefore, exploring *AS3MT* gene polymorphism will provide information on understanding susceptibility to pediatric epilepsy and drug safety.

## Data Availability Statement

The original contributions presented in the study are included in the article/Supplementary Material, further inquiries can be directed to the corresponding author/s.

## Ethics Statement

The studies involving human participants were reviewed and approved by Baoan Women’s and Children’s Health Hospital Ethics Committee. Written informed consent was obtained from the individual(s), and minor(s)’ legal guardian/next of kin, for the publication of any potentially identifiable images or data included in this article.

## Author Contributions

XF and HX: conceptualization. YC, JL, and HG: data curation. YC, JL, and WL: formal analysis. XF: funding acquisition. XF and XL: methodology. HX: supervision. YC, JL, and QC: project administration. XF and YC: writing—original draft. XF and HX: writing—review and editing. All authors contributed to the article and approved the submitted version.

## Conflict of Interest

The authors declare that the research was conducted in the absence of any commercial or financial relationships that could be construed as a potential conflict of interest.

## Publisher’s Note

All claims expressed in this article are solely those of the authors and do not necessarily represent those of their affiliated organizations, or those of the publisher, the editors and the reviewers. Any product that may be evaluated in this article, or claim that may be made by its manufacturer, is not guaranteed or endorsed by the publisher.

## Footnotes

1
http://string-db.org

2
https://www.genecards.org/

3
http://www.disgenet.org/

4
https://www.snpstats.net/start.htm

## Abbreviations

ADRs: adverse drug reactions
AEDs: antiepileptic drugs
AS3MT: arsenite methyltransferase
cADRs: cutaneous adverse drug reactions
CNNM2: cyclin and CBS domain divalent metal cation transport mediator 2
dADRs: digestive adverse drug reactions
LD: linkage disequilibrium
nADRs: nervous adverse drug reactions
OXC: oxcarbazepine
PCR: polymerase chain reaction
PPI: protein-protein interaction
SAMe: *S*-adenosyl -L-methionine
SNP: single nucleotide polymorphism
TRIM26: tripartite motif containing 26
VPA: valproic acid.
