# Supplementary material for: AS3MT Polymorphism: A Risk Factor for Epilepsy Susceptibility and Adverse Drug Reactions to Valproic Acid and Oxcarbazepine Treatment in Children From South China
Source: Front Neurosci. 2021 Nov 26;15:705297. doi: 10.3389/fnins.2021.705297 (PMC8661122; doi:10.3389/fnins.2021.705297)
Supplement: Supplementary file 1 [file Table_1.docx]

**SUPPLEMENTARY TABLES**

**TABLE S1.** Clinical and demographic characteristics of patients with cutaneous and nervous ADRs.

| **Characteristics** | **Patients with all AEDs treatment (n=174)** | | | | | |  | **Patients with VPA treatment (n=96)** | | | |  | **Patients with OXC treatment (n=49)** | | | |
| --- | --- | --- | --- | --- | --- | --- | --- | --- | --- | --- | --- | --- | --- | --- | --- | --- |
|  | **ADRs (n=123, %)** | **ADRs-No (n=51, %)** | **nADRs (n=58, %)** | **nADRs-No (n=116, %)** | **cADRs (n=30, %)** | **cADRs-No (n=144, %)** |  | **nADRs (n=36, %)** | **nADRs-No (n=60, %)** | **cADRs (n=13, %)** | **cADRs-No (n=83, %)** |  | **nADRs (n=13, %)** | **nADRs-No (n=36, %)** | **cADRs (n=7, %)** | **cADRs-No (n=42, %)** |
| **Age of diagnosis (years)** | 3.53 ± 3.05 | 3.61 ± 2.99 | 3.70 ± 3.11 | 2.45 ± 3.10 | 4.44 ± 3.55 | 3.32 ± 2.89 |  | 3.18 ± 2.86 | 3.56 ± 3.40 | 3.41 ± 2.71 | 3.32 ± 3.19 |  | 5.13 ± 3.53 | 3.70 ± 3.10 | 5.74 ± 4.75 | 3.71 ± 2.91 |
| ***p-*value** | 0.8364 | | 0.6179 | | 0.1145 | |  | 0.5596 | | 0.9133 | |  | 0.2115 | | 0.3101 | |
| **Gender** | | | | | | | | | | | | | | | | |
| Male | 69 (56.1%) | 29 (56.9%) | 30 (51.7%) | 68 (58.6%) | 15 (50.0%) | 82 (56.9%) |  | 16 (44.4%) | 39 (65.0%) | 9 (69.2%) | 37 (44.6%) |  | 6 (46.2%) | 14 (38.9%) | 1 (14.3%) | 19 (45.2%) |
| Female | 54 (43.9%) | 22 (43.1%) | 28 (48.3%) | 48 (41.4%) | 15 (50.0%) | 62 (43.1%) |  | 20 (55.6%) | 21 (35.0%) | 4 (30.8%) | 46 (55.4%) |  | 7 (53.8%) | 22 (61.1%) | 6 (85.7%) | 23 (54.8%) |
| ***p-*value** | 0.9262 | | 0.3872 | | 0.4860 | |  | 0.0487* | | 0.0980 | |  | 0.6478 | | 0.1229 | |
| **AEDs therapy** |  |  |  |  |  |  |  |  |  |  |  |  |  |  |  |  |
| Monotherapy | 71 (57.7%) | 39(76.5%) | 30(51.7%) | 82(70.7%) | 13(43.3%) | 99(68.7%) |  | 22(61.1%) | 28(46.7%) | 6(46.2%) | 41(49.4%) |  | 5(38.5%) | 18(50.0%) | 3(42.9%) | 21(50.0%) |
| Polytherapy | 52 (42.3%) | 12(23.5%) | 28(48.3%) | 34(29.3%) | 17(56.7%) | 45(31.3%) |  | 14(38.9%) | 32(53.3%) | 7(53.8%) | 42(50.6%) |  | 8(61.5%) | 18(50.0%) | 4(57.1%) | 21(50.0%) |
| ***p-*value** | 0.0196* | | 0.0138* | | 0.0082* | |  | 0.5019 | | 0.8278 | |  | 0.4749 | | 0.7263 | |
| **Causality assessment^#^** | |  |  |  |  |  |  |  |  |  |  |  |  |  |  |  |
| Certain | 10(3.7%) |  | 6(6.1%) |  | 2(4.4%) |  |  | 3(7.9%) |  | / |  |  | 2(10.5%) |  | 1(10.0%) |  |
| Probable | 232(85.9%) |  | 79(80.6%) |  | 41(89.1%) |  |  | 29(76.3%) |  | 16(88.9%) |  |  | 14(73.7%) |  | 9(90.0%) |  |
| Possible | 28(10.4%) |  | 13(13.3%) |  | 3(6.5%) |  |  | 6(15.8%) |  | 2(11.1%) |  |  | 3(15.8%) |  | / |  |

nADRs, nervous adverse drug reactions; cADRs, cutaneous adverse drug reactions.

*p < 0.05；

^#^ The number of ADRs observed in epileptic children receiving AEDs, which is higher than the number of patients in each group due to some patients presenting with more than one ADRs.

**TABLE S2.** Comparison of *AS3MT* rs7085104 diplotype distribution for epileptic children with or without nervous and cutaneous adverse drug reactions when received VPA.

| **ADRs** | **Genetic model** | **Diplotype** | **Patients with nADRs (n = 36)** | **Patients without nADRs (n = 60)** | **OR (95% CI)** | ***p-*value** |
| --- | --- | --- | --- | --- | --- | --- |
| **nADRs** | Allele contrast | A *vs.* G | 40 (55.6%) /  32 (44.4%) | 64 (53.3%) /  56 (46.7%) | 1.00  1.09 (0.61-1.98) | 0.76 |
|  | Codominant | AA *vs.* GA *vs.* GG | 12 (33.3%) /  16 (44.4%) /  8 (22.2%) | 17 (28.3%) /  30 (50.0%) /  13 (21.7%) | 1.00  1.32 (0.51-3.44)  1.15 (0.36-3.62) | 0.85 |
|  | Dominant | AA *vs.* GA + GG | 12 (33.3%) /  24 (66.7%) | 17 (28.3%) /  43 (71.7%) | 1.00  1.26 (0.52-3.09) | 0.61 |
|  | Recessive | AA + GA *vs.* GG | 28 (77.8%) /  8 (22.2%) | 47 (78.3%) /  13 (21.7%) | 1.00  0.97 (0.36-2.62) | 0.95 |
|  | Overdominant | AA + GG *vs.* GA | 20 (55.6%) /  16 (44.4%) | 30 (50.0%) /  30 (50.0%) | 1.00  1.25 (0.56-2.87) | 0.60 |
|  | Log-additive | AA *vs.* GG | 12 (33.3%) /  8 (22.2%) | 17 (28.3%) /  13 (21.7%) | 1.00  1.09 (0.61-1.94) | 0.77 |
| **ADRs** | **Genetic model** | **Diplotype** | **Patients with cADRs(n = 13)** | **Patients without cADRs (n = 83)** | **OR (95% CI)** | ***p-*value** |
| **cADRs** | Allele contrast | A *vs.* G | 11 (42.3%) /  15 (57.7%) | 92 (55.4%) /  74 (44.6%) | 1.00  0.59 (0.27-1.33) | 0.21 |
|  | Codominant | AA *vs.* GA *vs.* GG | 2 (15.4%) /  7 (53.8%) /  4 (30.8%) | 27 (32.5%) /  38 (45.8%) /  18 (21.7%) | 1.00  0.40 (0.08-2.09)  0.33 (0.06-2.01) | 0.40 |
|  | Dominant | AA *vs.* GA + GG | 2 (15.4%) /  11 (84.6%) | 27 (32.5%) /  56 (67.5%) | 1.00  0.38 (0.08-1.82) | 0.19 |
|  | Recessive | AA + GA *vs.* GG | 9 (69.2%) /  4 (30.8%) | 65 (78.3%) /  18 (21.7%) | 1.00  0.62 (0.17-2.26) | 0.48 |
|  | Overdominant | AA + GG *vs.* GA | 6 (46.2%) /  7 (53.8%) | 45 (54.2%) /  38 (45.8%) | 1.00  0.72 (0.22-2.34) | 0.59 |
|  | Log-additive | AA *vs.* GG | 2 (15.4%) /  4 (30.8%) | 27 (32.5%) /  18 (21.7%) | 1.00  0.60 (0.27-1.38) | 0.22 |

**TABLE S3.** Comparison of *AS3MT* rs7085104 diplotype distribution for epileptic children with or without nervous or cutaneous adverse drug reactions when received OXC.

| **ADRs** | **Genetic model** | **Diplotype** | **Patients**  **with nADRs**  **(n = 13)** | **Patients without nADRs**  **(n = 36)** | **OR (95% CI)** | ***p*-value** |
| --- | --- | --- | --- | --- | --- | --- |
| **nADRs** | Allele contrast | A *vs.* G | 14 (53.8%) /  12 (46.2%) | 40 (56.0%) /  32 (44.0%) | 1.00  0.93 (0.40-2.25) | 0.88 |
|  | Codominant | AA *vs.* GA *vs.* GG | 4 (30.8%) /  6 (46.1%) /  3 (23.1%) | 14 (38.9%) /  12 (33.3%) /  10 (27.8%) | 1.00  0.57 (0.13-2.51)  0.95 (0.17-5.23) | 0.72 |
|  | Dominant | AA *vs.* GA + GG | 4 (30.8%) /  9 (69.2%) | 14 (38.9%) /  22 (61.1%) | 1.00  0.70 (0.18-2.71) | 0.60 |
|  | Recessive | AA + GA *vs.* GG | 10 (76.9%) /  3 (23.1%) | 26 (72.2%) /  10 (27.8%) | 1.00  1.28 (0.29-5.64) | 0.74 |
|  | Overdominant | AA + GG *vs.* GA | 7 (53.9%) /  6 (46.1%) | 24 (66.7%) /  12 (33.3%) | 1.00  0.58 (0.16-2.12) | 0.42 |
|  | Log-additive | AA *vs.* GG | 4 (30.8%) /  3 (23.1%) | 14 (38.9%) /  10 (27.8%) | 1.00  0.95 (0.42-2.11) | 0.89 |
| **ADRs** | **Genetic model** | **Diplotype** | **Patients**  **with cADRs**  **(n = 7)** | **Patients without cADRs**  **(n = 42)** | **OR (95% CI)** | ***p*-value** |
| **cADRs** | Allele contrast | A *vs.* G | 8 (57.1%) /  6 (42.9%) | 46 (54.8%) /  38 (45.2%) | 1.00  1.10 (0.37-3.62) | 0.87 |
|  | Codominant | AA *vs.* GA *vs.* GG | 3 (42.9%) /  2 (28.6%) /  2 (28.6%) | 15 (35.7%) /  16 (38.1%) /  11 (26.2%) | 1.00  1.60 (0.23-10.94)  1.10 (0.16-7.74) | 0.88 |
|  | Dominant | AA *vs.* GA + GG | 3 (42.9%) /  4 (57.1%) | 15 (35.7%) /  27 (64.3%) | 1.00  1.35 (0.27-6.85) | 0.72 |
|  | Recessive | AA + GA *vs.* GG | 5 (71.4%) /  2 (28.6%) | 31 (73.8%) /  11 (26.2%) | 1.00  0.89 (0.15-5.25) | 0.90 |
|  | Overdominant | AA + GG *vs.* GA | 5 (71.4%) /  2 (28.6%) | 26 (61.9%) /  16 (38.1%) | 1.00  1.54 (0.27-8.89) | 0.62 |
|  | Log-additive | AA *vs.* GG | 3 (42.9%) /  2 (28.6%) | 15 (35.7%) /  11 (26.2%) | 1.00  1.08 (0.39-2.99) | 0.88 |
